# Exploring the association between social camouflaging and self- versus caregiver-report discrepancies in anxiety and depressive symptoms in autistic and non-autistic socially anxious adolescents

**DOI:** 10.1177/13623613241238251

**Published:** 2024-03-15

**Authors:** Jiedi Lei, Eleanor Leigh, Tony Charman, Ailsa Russell, Matthew J Hollocks

**Affiliations:** 1 King’s College London, UK; 2 South London and Maudsley NHS Foundation Trust, UK; 3 University of Oxford, UK; 4 University of Bath, UK

**Keywords:** adolescent, autism spectrum disorder, caregiver, depression, discrepancy, generalised anxiety, rater, social camouflaging

## Abstract

Social camouflaging in autism involves hiding social differences and autistic traits to fit in with neurotypical settings and is associated with poorer mental health in both autistic adolescents and adults. This study explored the association between self-reported social camouflaging behaviours and adolescents’ self-report of generalised anxiety disorder and depressive symptoms compared with caregiver reports. A clinical sample of 43 autistic and 39 non-autistic adolescents (14–19 years), without intellectual disability and matched on social anxiety, and their primary caregiver completed questionnaires reporting the young person’s autistic traits, generalised anxiety disorder and depression symptoms. Using response surface analysis, congruence between adolescent and caregiver rated autistic traits, generalised anxiety disorder and depression symptoms were not associated with greater camouflaging scores. Response surface analysis parameters showed that camouflaging was greater when both adolescent and caregivers rated high levels of autistic traits and generalised anxiety disorder symptoms, and when adolescents exceeded caregiver ratings on autistic traits, generalised anxiety disorder and depression symptoms. Adolescents who experience greater anxiety and autistic traits may engage in more (though less effective) social camouflaging behaviours, which in turn may contribute towards poorer mental health outcomes. Clinicians may benefit from collaboratively creating with adolescents a person-centred formulation that considers the associations between autistic traits and mental health outcomes.

**Lay abstract:**

Social camouflaging or masking refers to strategies autistic individuals adopt to hide their autism persona when trying to fit in. It is unclear whether camouflaging is only applicable to social differences unique to autism, or more generally to any types of social difference, such as experiences of mental health difficulties. We asked 43 autistic and 39 non-autistic adolescents (aged 14–19 years, all of whom showed similarly high levels of social anxiety) and their primary caregivers to complete questionnaires about their mental health (anxiety and depression) and autistic traits, and adolescents self-reported camouflaging behaviours. We wondered if camouflaging may be used to hide mental health difficulties reported by young people and affect caregiver report on symptom severity. We found that adolescents who self-reported greater levels of autistic traits, anxiety and depression symptoms compared with their caregivers reported greater camouflaging. Adolescents who agreed on having high levels of autistic traits and anxiety symptoms with their caregivers reported greater camouflaging behaviours. We discuss how having high levels of autistic traits and anxiety may increase adolescents’ camouflaging behaviours to hide social differences, which may contribute towards poor mental health outcomes. We think it is important to talk with adolescents about how camouflaging social and mental health difference can have negative impacts for mental health as well as possible positive social gains.

Autism spectrum disorder (ASD; hereon autism) is a neurodevelopmental disorder characterised by social communication differences, and restricted and repetitive patterns of behaviours and interests (American Psychiatric Association, 2022), with a national prevalence rate of 1 in 57 in school-aged children in England (Roman-Urrestarazu et al., 2021). Across development, systematic review and meta-analyses have found high levels of co-occurring mental health difficulties (Lai et al., 2019), with an overall anxiety diagnosis rate of around 42% (Simonoff et al., 2008), which is much higher than found in the general population (Gadow et al., 2005). Diagnostic rates of major depressive disorder and dysthymic disorder range from 0.5% to 0.9% in a population-derived cohort study of autistic children and adolescents aged 10–14 years (0.5%–0.9%) (Simonoff et al., 2008), to around 10.7% in a community-derived sample of autistic adolescents aged 13–17 years (Hollocks et al., 2022). Other studies have found between 15.8% and 54% of autistic adolescents to have co-occurring depression (Bitsika & Sharpley, 2015; Mayes et al., 2011; Uljarević et al., 2020), higher than the global 1-year prevalence for depression among neurotypical adolescents (8%) (Shorey et al., 2022). One scoping review evaluating depression in autistic young people aged 3–19 years found that the direction of causation between social difficulties and depression symptomatology is unclear and could be bidirectional, as awareness of social differences can increase vulnerability for depression, while depressive symptoms can reduce social motivation to interact with others (Kim & Lecavalier, 2021). A meta-synthesis of qualitative studies reviewing lived experiences of non-autistic adolescents with a diagnosis of unipolar depression reported hiding their symptoms from others to avoid social stigma or being bullied by others (Twivy et al., 2023), though it remains unclear whether the same pattern of masking behaviour can be seen among autistic adolescents who experience high levels of depressive symptoms.

Recent literature in autism has also talked about social masking (or social camouflaging), which encapsulates a range of behaviours from autistic individuals hiding their autism symptoms to portray a non-autistic persona to avoid social judgement (e.g. ‘I am always aware of the impression I make on other people’), adopting behavioural strategies to actively compensating for difficulties in social situations (e.g. ‘I practise my facial expression and body language to make sure they look normal’) as well as trying to fit in with neurotypical peers through assimilation (e.g. ‘In social situations, I feel like I’m pretending to be ‘normal’’) (Hull et al., 2017, 2019). Autistic individuals describe camouflaging as a way to reduce social stigma and bullying from others by hiding one’s autistic traits (Pearson & Rose, 2021; Schneid & Raz, 2020).

The concept of altering one’s presentation of self to come across well in a social interaction goes beyond camouflaging in the context of autism and is conceptually consistent with impression management behaviours (Ai et al., 2022; Goffman, 1959). The ability for individuals to actively monitor the presentation of self in social contexts and adjust one’s behaviours accordingly may decrease discrimination and shame, by hiding undesired characteristics and stigmatised identities away from the public eye (Ai et al., 2022). However, the stress of camouflaging over time is thought to, in some cases, lead to increased burnout and feeling inauthentic in one’s view of oneself (Bargiela et al., 2016; Mandy, 2019; Schneid & Raz, 2020). Hiding one’s autism identity and experience of internalised stigma is associated with poorer mental health outcomes (Botha & Frost, 2020), and may inhibit autistic individuals from finding a positive sense of self-worth and developing collective self-esteem to build a shared autism group identity and to recognise and embrace their own strengths within their social group (Cage et al., 2018; Cooper et al., 2017, 2022). In a recent mixed-methods systematic review on social masking (in the context of social camouflaging in the autism literature), authors found that the consequences of social camouflaging included delays in receiving diagnosis and timely support that contribute towards burnout, lower self-esteem and increased identity confusion (Zhuang et al., 2023).

Adolescence marks a developmental phase characterised by a heightened awareness and sensitivity to peer rejection while being thrusted into ever changing social environments (Blakemore & Robbins, 2012; Casey et al., 2008). Adolescents are motivated to develop one’s own social identity and gain peer approval (Blakemore & Robbins, 2012). Comparing autistic adolescents to neurotypical peers between the ages of 13 and 18 years, one study found that older adolescents (16–18 years) reported more camouflaging compared with younger peers in the neurotypical group only (Jorgenson et al., 2020). Interestingly, neurotypical adolescents reported more masking behaviours compared with autistic peers, whereas autistic adolescents reported greater assimilation behaviours (Jorgenson et al., 2020), suggesting that where neurotypical peers may hide one’s perceived social differences to maintain relationships, autistic peers may need to work harder to actively ‘appear normal’ and fit in with peers.

Elevated camouflaging reported by autistic and neurotypical adolescents is associated with greater anxiety and depression symptoms (Bernardin, Mason, et al., 2021; Ross et al., 2023), suggesting masking one’s authentic self when socialising with others may come at a cost to all adolescents regardless of autism diagnosis (Bernardin, Lewis, et al., 2021). Authenticity is related to many aspects of adaptive functioning, including more positive relationship functioning, higher self-esteem and greater positive affect (Brunell et al., 2010; Goldman & Kernis, 2002; Heppner et al., 2008; Robinson et al., 2013; Wood et al., 2008), thus masking one’s authenticity during adolescence may increase vulnerability to developing internalising conditions. Many adolescents attempt to appear ‘normal’ and fit in with peers, and this may involve camouflaging of not just autism-related social differences, but also mental health difficulties like anxiety and depression. Furthermore, it is unclear whether autism symptom severity may interfere with the perceived success of camouflaging in the eyes of observers (i.e. caregivers or teachers), especially if camouflaging is used to mask broader mental health difficulties beyond social communication differences.

One long-standing source of discrepancy in measurements of internalising symptoms in autistic adolescents is found between self and caregiver reports. Although collating information from multiple informants on internalising symptoms in childhood is considered best practice (Hunsley & Marsh, 2007), a meta-analysis found that the correlation between caregiver and young person report of childhood internalising symptoms is relatively weak (*r* = 0.25) in the general population (De Los Reyes et al., 2015; De Los Reyes & Kazdin, 2005). Among autistic adolescents, findings suggest mixed reports of agreement on anxiety ratings provided by caregivers and adolescents (Blakeley-Smith et al., 2012; May et al., 2015; Ozsivadjian et al., 2014; Storch et al., 2012), and discrepancies are also inconsistent in direction, with some suggesting parents report higher levels of anxiety than adolescents (Bitsika & Sharpley, 2015; Blakeley-Smith et al., 2012; Ozsivadjian et al., 2014) and others suggesting the reverse (Hurtig et al., 2009; Magiati et al., 2014).

Previous studies have suggested that parents may conflate symptoms of anxiety with autism in the context of individual differences in social communication and accurate emotion expression, and may be better at rating clinically significant levels that are more observable (Kalvin et al., 2020). No studies to date have explored to what extent caregiver–child discrepancies in rating internalising symptoms may be associated with adolescents’ efforts to camouflage their perceived social differences, and whether there may be differences between autistic and non-autistic adolescents. It may be that adolescents who experience greater internalising symptoms increase their self-monitoring and impression management when interacting with others, and the success of their camouflaging may influence caregivers’ perception of the extent of their anxiety and depression symptoms, who often serve a mediating role in initiating service contact for professional support.

The current study had three aims using a clinical sample of autistic and non-autistic adolescents who are accessing mental health services and matched on symptom severity of social anxiety using self-report measures. The first aim is to investigate differences in caregiver report and adolescent self-reports on symptoms of autistic traits, social anxiety, depression and generalised anxiety. We hypothesise that adolescents will report greater severity of symptoms of generalised anxiety and depression, and fewer autistic traits, when compared with caregiver reports. The second aim is to investigate the association between camouflaging behaviours and symptom severity of generalised anxiety and depression as reported by adolescents. We hypothesise that greater camouflaging behaviours will be associated with greater symptom severity of generalised anxiety and depression symptoms as reported by adolescents. The third aim is to explore the association between camouflaging behaviours and discrepancy in ratings between adolescents’ self-report and caregiver reports of generalised anxiety and depression symptoms, when accounting for autistic traits. We hypothesise that greater camouflaging behaviours will be associated with greater self- versus caregiver-report discrepancy in symptoms of generalised anxiety and depression.

## Method

### Participants

A total of 82 adolescents (14–19 years old) and their nominated caregivers (parent or carer) were recruited from the Child and Adolescent Mental Health Services (CAMHS) in South London. Lists of autistic and non-autistic adolescents potentially eligible for the study were generated via records held at the National Institute of Health and Care Research South London and Maudsley NHS Foundation Trust Biomedical Research Centre’s Consent for Contact scheme (C4C), and each participant was contacted via telephone or email with study information and asked to take part in the study. Clinical diagnosis of ASD or equivalent by a qualified professional for autistic adolescents (*n* = 43) was gathered either from clinical records from their local CAMHS access or provided by caregiver electronically. Adolescents in the non-autism group (*n* = 39) did not have any clinical diagnosis of autism via self- nor caregiver report, and none were recorded on their CAMHS records. Exclusion criteria for both groups included having a formal clinical diagnosis of intellectual disability, epilepsy, genetic or psychotic conditions, have an active safeguarding concern on current clinical records, is residing in an in-patient unit at the time of data collection or non-fluent in written English.

### Measures

A detailed summary of all demographic and outcome measures is provided in Supplementary Materials – Methods section. At the point of initial contact, non-autistic adolescents or their caregiver completed the 3-item Mini-SPIN (Connor et al., 2001) to screen for high levels of social anxiety that matched the autistic adolescent group. All adolescents and their caregivers completed demographic questions including age, gender, identity, ethnicity and socioeconomic status. All adolescents completed the Receptive One-Word Picture Vocabulary Test (4th Edition, Martin & Brownell, 2010) to assess English reading ability. Adolescents completed Autism Quotient-28 to measure autistic traits, Social Phobia Inventory (SPIN; Johnson et al., 2006) to assess social anxiety symptoms, Camouflaging Autistic Traits Questionnaire (CAT-Q; Hull et al., 2019), and Revised Children’s Anxiety and Depression Scale – Depression and Generalised Anxiety subscale (RCADS-Dep, RCADS-GAD; Baron et al., 2021) to measure symptoms of generalised anxiety disorder (GAD) and depression, with T-score ⩾ 70 meeting clinical cut-off (Esbjørn et al., 2012). Caregivers completed Autism Quotient-Adolescent version to assess autistic traits and RCADS-Parent Depression and GAD subscales (Baron et al., 2021) for evaluating symptom severity in their young person.

### Procedure

Participants in the current study were recruited for a larger study (Lei et al., 2023) that examined the association between camouflaging in autism and safety behaviours (i.e. avoidance of social situations or managing how one comes across in social interactions) in social anxiety in autistic and non-autistic adolescents. The original study and matched participant groups based on social anxiety symptom severity, with an aim to explore the association between autistic traits, camouflaging and safety behaviours when accounting for social anxiety symptom severity. Based on previous studies that suggested autistic adolescents experience greater social anxiety symptoms than non-autistic peers, screening was completed for non-autistic adolescents using the 3-item Mini-SPIN (Connor et al., 2001) for high levels of social anxiety symptoms. At the point of initial contact, non-autistic adolescents or their caregiver was asked to complete the Mini-SPIN with those scoring 6 or higher invited to take part in the full questionnaire session. Adolescents who met study eligibility criteria used a link to access the full questionnaire session on Qualtrics, where they first read through the study information sheet, provided written assent (aged 14–15 years, with caregivers providing written consent) or consent (aged 16–19 years) depending on their age, before completing demographic information and questionnaires. Participants also completed a one-word reading task on Gorilla to assess their reading ability. Adolescents who successfully completed the full session were reimbursed £5 in gift vouchers to compensate for their time.

### Ethical approval

All procedures in the current study comply with the ethical standards of the relevant national and institutional committees on human experimentation and with the Helsinki Declaration of 1975 (revised in 2008). All procedures involving human subjects/patients were approved by the NHS Health Research Authority.

### Community involvement

As part of Patient and Public Involvement, one autistic young person accessing mental health support from CAMHS was recruited to review the research information and consent forms, as well as questionnaire forms to check that the information and language use were appropriate and clear, and that the questionnaire completion time was feasible.

### Analyses

A detailed summary of the analysis plan is found in the Supplementary Materials. All statistical analyses were completed in SPSS v.28, and response surface analysis (RSA) was completed in R using RSA package (Schönbrodt & Humberg, 2023). All participants completed all measures with no missing data. First, descriptive statistics and between-group differences for both caregiver and adolescent demographic variables were completed using independent sample *t*-tests and chi-square tests. Second, four independent two-way ANOVAs were completed to assess differences in ratings of autistic traits, symptoms of depression, generalised anxiety and social anxiety by rater (adolescent vs caregiver), and by diagnostic group (autism vs non-autism), with Benjamini–Hochberg procedure to control for false discovery rate and multiple comparisons, and report adjusted *p* values. Assuming a small-medium effect size (*f* = 0.2; Cohen, 1988) and 80% power, *a priori* sample size calculation using G*Power suggested a sample size of 72 (36 in each of the two groups) would be required to observe statistically significant effect (*p* < 0.05) for an interaction effect. We generated Bland–Altman plots to visually depict interrater agreement between adolescents and caregivers on symptom ratings for depression and GAD (Bland & Altman, 1995).

Third, we completed partial correlations (controlling for age, gender – using male vs other gender identities as binary dummy coding) to explore the association between camouflaging behaviours and individual differences in symptom severity for autistic traits, generalised anxiety and depression as reported by adolescents, using Benjamin–Hochberg to control for multiple comparisons. Fourth, we completed RSA to explore to what extent assumed similarity in ratings of autistic traits, generalised anxiety and depression severity ratings by adolescents and their caregivers are associated with total camouflaging scores. After centring each predictor on the questionnaire’s midpoint, we first added age and gender (male vs other identities) as covariates (and autistic traits for models on GAD and depression symptoms). We included the following predictors: (1) adolescent’s self-report symptom severity (X), (2) caregiver’s report of adolescent’s symptom severity (Y), (3) the quadratic term of adolescent’s self-report (X^2^), (4) the interaction between adolescent and caregiver report (X*Y), and (5) the quadratic term of caregiver’s report (Y^2^). We chose the RSA approach over hierarchical linear regressions as it allows us to model linear and curvilinear relationship at *different levels* of both *matches and mismatches* between different predictors with the outcome variable (Barranti et al., 2017). We first report interpretation of analysis of lines of congruence and incongruence generated from the RSA as outlined by Barranti et al. (2017). However, to avoid over-interpretation of the RSA parameters when considered in isolation, we follow the four-step checklist from Humberg et al. (2019) to examine the congruence hypothesis for each analysis by combining outcomes from the lines of congruence, incongruence and first principal axis. The congruence hypothesis states that the outcome variable (i.e. camouflaging) is higher the closer together (i.e. congruent) the adolescent and parent ratings are to one another.

## Results

Participant demographic variables are summarised in Table 1 for caregiver (Table 1) and adolescents (Table 2). Caregivers between the two diagnostic groups did not differ in age (*t* (80) = .91, *p* = 0.37), gender (*Χ*^2^ (1) = 0.33, *p* = 0.57), ethnicity (*Χ*^2^ (3) = 2.37, *p* = 0.50), and education status (*Χ*^2^ (1) = 1.98, *p* = 0.16). For employment status, caregivers of autistic adolescents had a greater employment rate than those in the non-autism group (*Χ*^2^ (1) = 5.67, *p* = 0.02). Adolescents between the two diagnostic groups did not differ in age (*t* (80) = 0.80, *p* = 0.42), gender identity (*Χ*^2^ (2) = 1.19, *p* = 0.55), reading ability based on standardised scores (*t* (69) = −0.34, *p* = 0.37) or eligibility for free school meals (*Χ*^2^ (2) = 0.3, *p* = 0.86). For social camouflaging reported by adolescents, no between-group differences were found for compensation (*t* (80) = −1.30, *p* = 0.20), assimilation (*t* (80) = −0.91), *p* = 0.69) or masking (*t* (80) = 1.59, *p* = 0.12). All effect sizes are reported in Table 3. One sample *t*-test showed that the current sample scores are significantly higher than those found in a UK clinical sample of young people aged 8–18 years (*n* = 1920), who presented to mental health services with mild to moderate difficulties (Depression (*M*) = 11.05, *t*(81) = 8.09, *p* < 0.001; GAD (*M*) = 8.04, *t*(81) = 5.96, *p* < 0.001) (Baron et al., 2021), suggesting the samples scored within a clinical range on the RCADS. Comparison of CAT-Q and social anxiety scores between the current sample and other studies from the general population are shown in Supplementary Materials Table 1.

**Table 1.** Participant demographic information for autism and non-autism groups: Caregiver (parent/carer) information.

	Autism (*n* = 43) *M* (*SD*; range)	Non-autism (*n* = 39) *M* (*SD*; range)
Age (years)	48.33 (6.29; 36–58)	49.72 (7.57; 32–66)
Gender	*n* (%)	
Male	2 (4.65)	3 (8.33)
Female	41 (95.35)	36 (92.31)
Ethnicity		
White	35 (81.40)	32 (82.05)
Black	3 (6.98)	4 (10.26)
Asian	0	1 (2.56)
Mixed/other	5 (11.63)	2 (5.13)
Parent/carer qualification		
Degree level of equivalent	29 (67.44)	21 (53.85)
Below degree level	11 (25.58)	15 (38.46)
No qualifications/prefer not to say	3 (6.98)	3 (7.69)
Job (employed)	42 (97.67)	32 (82.05)
Socioeconomic status (SES)		
Considers self from lower SES	6 (13.95)	4 (10.26)
Compared with others – from lower SES	9 (20.93)	4 (10.26)

**Table 2.** Participant demographic information for autism and non-autism groups: Adolescent information.

	Autism (*n* = 43) *M* (*SD*; range)	Non-autism (*n* = 39) *M* (*SD*; range)
Age (years)	15.70 (1.55; 14–19)	15.97 (1.56; 14–19)
Reading task	(*n* = 37)	(*n* = 34)
Raw score	152.51 (13.48; 118–177)	153.82 (14.26; 106–175)
Standard score	111.38 (12.81; 85–145)	112.5 (14.69; 73–145)
Social camouflaging (CAT-Q)	(*n* = 43)	(*n* = 39)
Assimilation	39.91 (9.7; 12–56)	38 (9.15; 9–53)
Compensation	35.86 (15.12; 11–63)	31.82 (12.80; 9–60)
Masking	36.26 (11.10; 8–52)	39.69 (8.02; 19–54)
Total	112.02 (32; 48–169)	109.51 (24.68; 48–159)
Gender	*n* (%)	
Male	13 (30.23)	8 (20.51)
Female	25 (58.14)	27 (69.23)
Non-binary/other	5 (11.63)	4 (10.26)
Ethnicity		
White	34 (79.07)	29 (74.36)
Black	3 (6.98)	5 (12.82)
Asian	0	1 (2.56)
Mixed/other	6 (13.95)	4 (10.26)
School		
State	27 (62.79)	35 (89.74)
Private–bursary	4 (9.30)	1 (2.56)
Private – full fee	5 (11.63)	3 (7.69)
Home-schooled	1 (2.33)	0
Prefer not to say/other	6 (13.95)	0
Living in care	1 (2.33)	2 (5.13)
Mental health conditions		
ADHD	7 (16.28)	0
Generalised anxiety disorder	23 (53.49)	8 (20.51)
Social anxiety disorder	2 (4.65)	2 (5.13)
Obsessive compulsive disorder	17 (39.53)	13 (33.33)
Panic	1 (2.33)	1 (2.56)
Post-traumatic stress disorder	1 (2.33)	2 (5.13)
Depression	12 (27.91)	6 (15.38)
Eating disorder	1 (2.33)	5 (12.82)

ADHD: attention deficit hyperactivity disorder; CAT-Q: Camouflaging Autism Traits Questionnaire; SES: socioeconomic status.

**Table 3.** Results of ANOVAs with measures of autism traits, social anxiety, generalised anxiety and depression as dependent variables.

	Autism (*n* = 43) *M* (*SD*; range)	Non-autism (*n* = 39) *M* (*SD*; range)	Row *d′_z_*	Effect type (raw score)	*F* (1, 80)	*p-*value	Partial η^2^
Autism traits							
AQ – 50 (Caregiver)	32.35 (5.98; 19–44)	23.13 (8.92; 10–40)	1.21	Main group	20.96	<0.001**	0.21
	*n* (%) > cut-off: 28 (65%)	*n* (%) > cut-off: 8 (21%)					
AQ – 28 (YP)	76.14 (10.21; 51–100)	70.31 (11.87; 39–98)	0.53	Main rater	0.13	0.91	<0.001
	*n* (%) > cut-off: 33 (69%)	*n* (%) > cut-off: 23 (59%)					
Column *d′_z_*	0.32	0.27		Interaction	6.04	0.016*	0.07
Social anxiety							
RCADS-P (Caregiver)	16.51 (6.61; 0–27)	16.38 (6.02; 7–27)	0.02	Main group	0.001	0.97	<0.001
SPIN Total (YP)	37.49 (15.40; 4–63)	37.59 (13.95; 8–61)	0.01	Main rater	<0.001	0.99	<0.001
Column *d′_z_*	0.01	0.01		Interaction	0.01	0.92	<0.001
Depression			Row *d′* (Raw)				
RCADS-P (Caregiver)	Raw:14.33 (6.55; 2–28) T (*n* = 38): 78.66 (20.63; 41–123) *n* (%) T > cut-off: 24 (56%)	Raw: 13.03 (5.77; 3–27) T (*n* = 35): 76.34 (18.71; 44–121) *n* (%) T > cut-off: 21(54%)	0.21	Main group	1.49	0.23	0.02
RCADS-C (YP)	Raw: 18.3 (7.25; 4–30) T (*n* = 38): 76.08 (19.52; 41–111) *n* (%) T > cut-off: 24 (56%)	Raw: 16.46 (6.98; 1–27) T (*n* = 35): 70.23 (18.68; 32–103) *n* (%) T > cut-off: 19 (49%)	0.26	Main rater	26.11	<0.001**	0.25
Column *d′* (Raw)	0.57	0.54		Interaction	0.14	0.71	0.002
Generalised anxiety							
RCADS-P (Caregiver)	Raw: 8.84 (3.92; 1–18) T (*n* = 38): 69.32 (16.05; 38–112) *n* (%) T > cut-off: 16 (37%)	Raw: 9.59 (4.40; 2–18) T (*n* = 35): 71.71 (16.36; 45–109) *n* (%) > cut-off: 17 (44%)	0.18	Main group	2.04	0.16	0.03
RCADS-C (YP)	Raw: 10.26 (4.48; 1–18) T (*n* = 38): 57.79 (12.48; 29–81) *n* (%) T > cut-off: 6 (14%)	Raw: 11.92 (4.57; 2–18) T (*n* = 35): 60.91 (14.20; 35–87) *n* (%) T > cut-off: 11 (28%)	0.37	Main rater	17.10	<0.001**	0.18
Column *d′* (Raw)	0.34	0.52		Interaction	1.02	0.32	0.01

*Note.* AQ = Autism Quotient, AQ-50 cut-off at > 30 and AQ-28 cut-off at ⩾ 70; Revised Children’s Anxiety and Depression Scale – Depression and Generalised Anxiety Subscales (Parent and Child). Note that T-score is only calculated for those who identified as either male or female (ASD: *n* = 38; Non-ASD: *n* = 35). Clinical threshold is for T-score ⩾ 70; Social Phobia Inventory (Child). Cohen’s *d*′ and *d*′_*z*_ are effect sizes for raw and standardised scores. *p_adjusted_ < 0.05, **p_adjusted_ < 0.01 (after using Benjamini–Hochberg corrections).

### Reporter differences in symptom severity for autistic traits, social anxiety, generalised anxiety and depression

**Figure 1.**
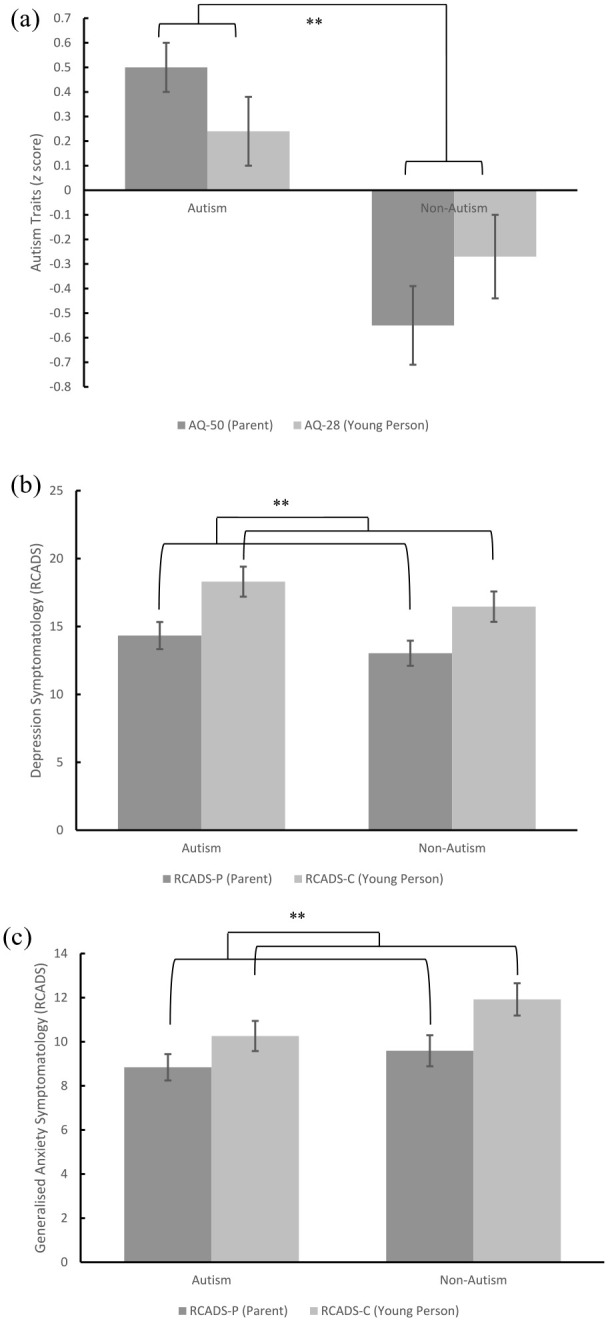
Graphical representation of group and rater differences in (a) autism traits; (b) depression symptomatology; and (c) generalised anxiety symptomatology. *Note.* AQ: Autism Quotient; RCADS-P/C: Revised Children’s Anxiety and Depression Scale – Depression and Generalised Anxiety Subscales (Parent and Child); SPIN: Social Phobia Inventory; **p_adjusted_ < 0.01 (using Benjamini–Hochberg corrections).

Results for the four ANOVAs including effect sizes are listed in Table 3, and significant group or rater differences are shown in Figure 1. For the first ANOVA with autistic traits as the dependent variable, a significant main effect of group was found (*F* (1, 80) = 20.96, *p*_adjusted_ = 0.004, partial η^2^ = 0.21), and post hoc comparisons found that autistic adolescents scored higher than those in non-autism group (*t* (80) = 0.78, *p*_adjusted_ < 0.001). No main effect of rater (*p*_adjusted_ = 0.99) was found. A significant group by rater interaction (*p*_adjusted_ = 0.048) was found, as both caregivers (*t*(80) = 1.05, *p* < 0.001) and young people (*t*(80) = 0.51, *p*_adjusted_ = 0.02) in the autism group rated autistic traits higher than those in the non-autism group. For the second ANOVA with social anxiety symptoms as the dependent variable, given that the two groups were matched on symptom severity of social anxiety, no main effect of rater (*p*_adjusted_ = 0.99), group (*p*_adjusted_ = 0.99) or rater by group interactions (*p*_adjusted_ = 0.99) were found.

**Figure 2.**
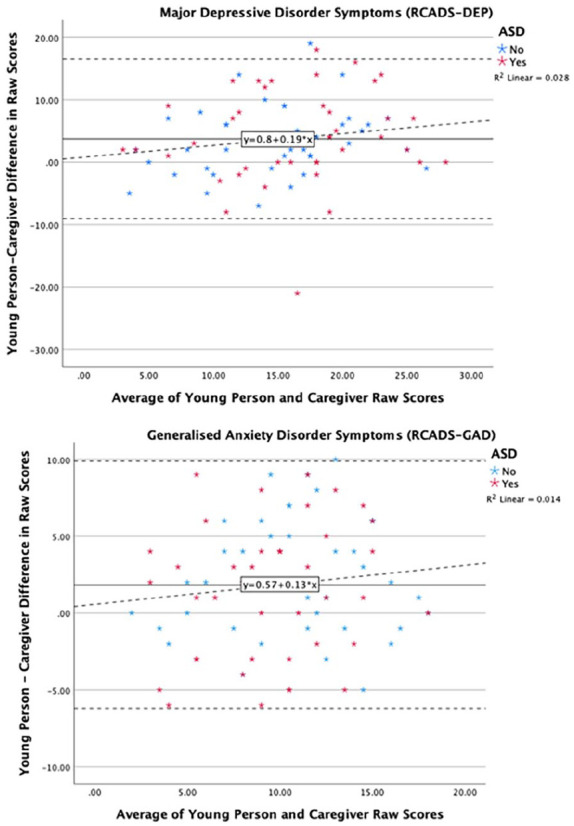
Bland–Altman plots showing average agreement between young person and caregiver reported generalised anxiety and depressive symptoms. *Note.* For each plot, the *y*-axis shows the difference in raw scores between adolescent and caregiver reports, and the *x*-axis shows the average of the adolescent and caregiver raw scores. Within each plot, the average difference score (young person – caregiver score) is represented by a solid black line and 95% confidence intervals are represented by dashed grey lines above and below the average score line. A positive difference score indicates that for that individual adolescent, the adolescent report is higher than the caregiver report, and vice versa. The association between the difference score and the mean average score is represented by the dashed black line.

For the third ANOVA with depression symptomatology (raw scores on RCADS) as the dependent variable, a significant main effect of rater was noted (*F* (1, 80) = 26.11, *p*_adjusted_ = 0.004, partial η^2^ = 0.25), and post hoc comparisons using Bonferroni corrections found that adolescents rated significantly higher levels of depressive symptoms than caregivers (*t* (80) = 3.71, *p*_adjusted_ < 0.001). No main effect of group (*p*_adjusted_ = 0.46) or group by rater interaction (*p*_adjusted_ = 0.99) was found. For the fourth ANOVA with generalised anxiety symptomatology (raw scores on RCADS) as the dependent variable, a significant main effect of rater was noted (*F* (1, 80) = 17.1, *p*_adjusted_ = 0.004, partial η^2^ = 0.18), and post hoc comparisons using Bonferroni corrections found that adolescents rated significantly higher levels of generalised anxiety than caregivers (*t* (80) = 1.88, *p*_adjusted_ < 0.001). No main effect of group (*p*_adjusted_ = 0.38) or group by rater interaction (*p*_adjusted_ = 0.55) was found. To visualise informant agreement and variation in agreement by score severity, Bland–Altman plots were generated as shown in Figure 2. No significant relationships between the difference scores and the average combined scores were found for depressive symptoms (*β* = 0.17, *p* = 0.13), nor symptoms of GAD between adolescent and caregiver reports (*β* = 0.12, *p* = 0.29).

### Associations between adolescent’s self-report symptoms and social camouflaging

Table 4 shows that when controlling for age and gender, camouflaging total and subscale scores rated by adolescents across the whole sample showed strong positive associations (*r* = 0.41 to 0.92, *p*_adj_ < 0.01), and autistic traits were significantly associated with all camouflaging subscales (*r* = 0.46 to 0.62, *p*_adj_ < 0.01) except for masking (*r* = 0.13, *p*_adj_ = 0.25). Both generalised anxiety and depression symptoms rated by adolescents were significantly associated with all camouflaging scales (*r* = 0.27 to 0.51, *p*_adj_ < 0.05), and with each other (*r* = 0.50, *p*_adj_ < 0.01). However, only greater self-reports of depression symptoms were significantly associated with greater autistic traits (*r* = 0.26, *p*_adj_ = 0.02), not generalised anxiety symptoms (*r* = 0.11, *p*_adj_ = 0.33). Table 5 shows that patterns of significant associations remained the same when also controlling for autistic traits across the whole sample.

**Table 4.** Partial correlations to show associations between adolescent’s self-report of autistic traits, generalised anxiety, depression and social camouflaging in the total sample (*N* = 82) when controlling for: Age, gender (male vs other) (*df* = 78).

	CAT-Q total	CAT-Q compensation	CAT-Q masking	CAT-Q assimilation	AQ-28 total	GAD (RCADS)
CAT-Q compensation	0.92**					
CAT-Q masking	0.83**	0.68**				
CAT-Q assimilation	0.76**	0.58**	0.41**			
AQ-28 total	0.48**	0.46**	0.13	0.62**		
GAD (RCADS)	0.38**	0.27*	0.32**	0.40**	0.11	
DEP (RCADS)	0.47**	0.36**	0.35**	0.51**	0.26*	0.50**

**Table 5.** Partial correlations to show associations between adolescent’s self-report of autistic traits, generalised anxiety, depression and social camouflaging in the total sample (*N* = 82) when controlling for age, gender (male vs other) and autistic traits (AQ) (*df* = 77).

	CAT-Q total	CAT-Q compensation	CAT-Q masking	CAT-Q assimilation	GAD (RCADS)
CAT-Q compensation	0.90**				
CAT-Q masking	0.88**	0.70**			
CAT-Q assimilation	0.68**	0.42**	0.43**		
GAD (RCADS)	0.38**	0.25*	0.31**	0.43**	
DEP (RCADS)	0.41**	0.28*	0.33**	0.47**	0.49**

CAT-Q: Camouflaging Autistic Traits Questionnaire; DEP: depression; GAD: generalised anxiety disorder; RCADS: Revised Children’s Anxiety and Depression Scale.

* *p_adjusted_* < 0.05. **p_adjusted_ < 0.01 (using Benjamini–Hochberg corrections).

### Association between assumed similarity in autistic traits and social camouflaging

**Figure 3.**
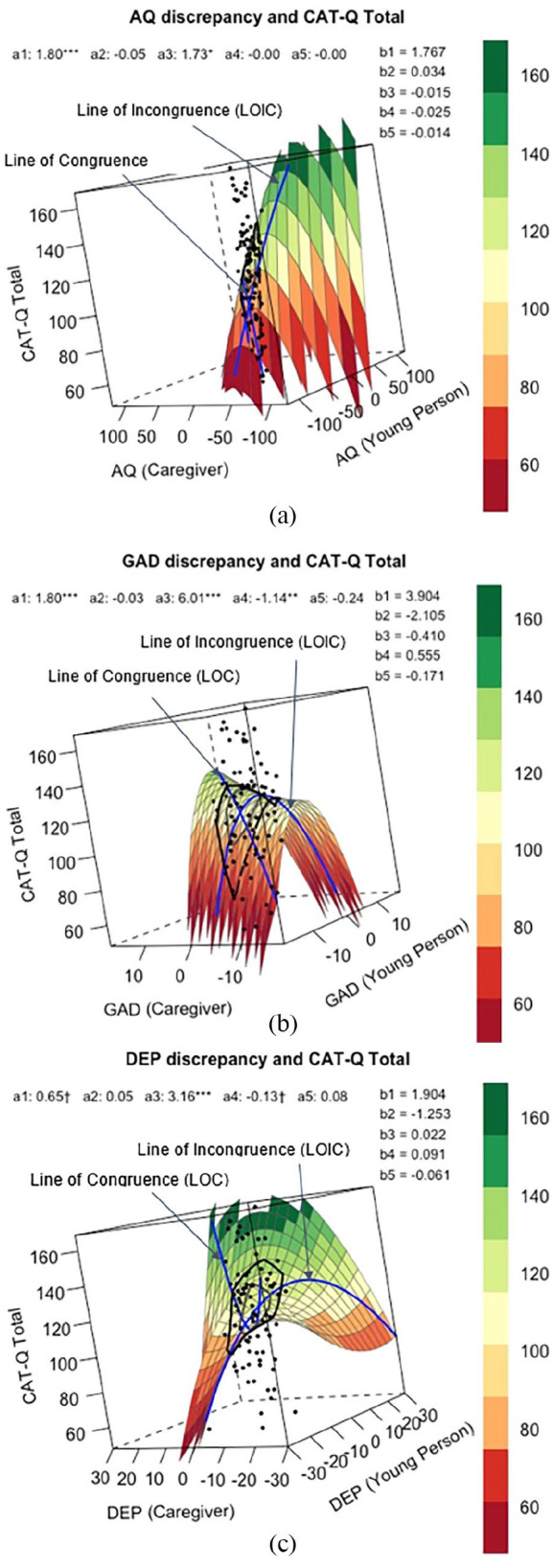
Response surface analysis for assumed similarity of (a) autistic traits, (b) generalised anxiety symptom severity and (c) depression symptom severity, as rated by young person (predictor X) and caregiver (predictor Y) when predicting social camouflaging behaviours (outcome Z). *Note. X* and *Y* values of 0 reflect the midpoint of the scale, and legend shows the corresponding outcome *Z* values. The line of congruence (*a_1_* = slope, *a_2_* = curvature) shows cases where *X* and *Y* perfectly match, and line of incongruence (*a_3_* = slope, *a_4_* = curvature) where *X* are the opposites of *Y* values (both shown in blue). Bagplot (black lines superimposed on response surface) shows the prevalence of different combinations of *X* and *Y* predictors. More information on polynomial coefficients (*b* values; *b_0_* = intercept, *b_1_* = *X*, *b_2_* = *Y*, *b_3_* = *X*^2^, *b_4_* = X*Y, *b_5_* = *Y*^2^) is reported in Tables 6 and 7. *p values here are unadjusted for multiple comparisons.

Figure 3(a) shows how all combinations of adolescent (X) and caregiver (Y) reports of autistic traits related to adolescent’s report of total camouflaging behaviours (Z), when controlling for age and gender (male vs other gender identities), and all polynomial coefficients are reported in Tables 6 and 7. Analysing the line of congruence, camouflaging was significantly greater when adolescent and caregiver rated autistic traits matched at higher levels than at lower levels (*a*_1_ = 1.80, *p* < 0.001), though it was not significantly associated with how adolescent and caregiver rated autistic traits matched at either midrange or extreme levels (*a*_2_ = −0.054, *p* = 0.195). Analysing the line of incongruence, social camouflaging was significantly greater when adolescent rated autistic traits exceeded caregiver ratings (*a*_3_ = 1.73, *p* = 0.03). Camouflaging was not associated with the extent to which adolescent and caregiver rated autistic traits matched or deviated from each other (*a*_4_ = −0.001, *p* = 0.93). When analysing the line of congruence, incongruence and first principal axis together, the RSA output did not support the congruence hypothesis. In other words, camouflaging was not greater when adolescent and parent-rated autism traits were closer to one another, as *a*_3_ (slope along the line of incongruence) was significantly different from 0 (*a*_3_ = 1.73, *p* = 0.029).

**Table 6.** Response surface for assumed similarity of autistic traits, generalised anxiety and depression scores reported by young person and caregiver in relation to total social camouflaging behaviours reported by young person in the total sample (*N* = 82): Estimated regression model.

	*B* (*SE*)	95% CI	*β*	*p-*value	*R*^2^ *(p-*value)
Model 1 (Autistic traits)					0.36 (<0.001)
Covariates					
Age	−0.36 (1.52)	−3.34, 2.62	−0.02	0.81	
Gender	16.26 (5.84)	4.81, 27.72	0.25	0.005*	
Predictors					δ*R*^2^ *(p* value): 0.26 (<0.001)
* b*_0_	90.27 (6.02)	78.47, 102.06	3.18	<0.001**	
* b_1_*–YP	1.77 (0.39)	1.00, 2.53	0.70	<0.001**	
* b_2_*–C	0.03 (0.48)	−0.91, 0.97	0.01	0.94	
* b_3_*–YP^2^	−0.02 (0.01)	−0.04, 0.01	−0.21	0.16	
* b_4_*–YP x C	−0.03 (0.02)	−0.07, 0.02	−0.16	0.27	
* b_5_*–C^2^	−0.01 (0.04)	−0.01, 0.07	−0.04	0.74	
Model 2 (Generalised anxiety)					0.47 (<0.001)
Covariates					
Age	−1.20 (1.34)	−3.82, 1.43	−0.07	0.37	
Gender	9.89 (5.42)	−0.73, 20.51	0.15	0.07	
AQ	1.11 (0.22)	0.69, 1.53	0.44	<0.001**	
Predictors					δ*R*^2^ (*p* value): 0.16 (0.001)
* b*_0_	110.32 (4.36)	101.76, 118.87	3.88	<0.001**	
* b_1_*–YP	3.90 (0.67)	2.58, 5.23	0.63	<0.001**	
* b_2_*–C	−2.11 (0.64)	−3.35, –0.86	−0.31	<0.001**	
* b_3_*–YP^2^	−0.41 (0.14)	−0.69, −0.14	−0.38	0.003**	
* b_4_*–YP × C	0.56 (0.21)	0.15, 0.96	0.40	0.007*	
* b_5_*–C^2^	−0.17 (0.16)	−0.48, 0.14	−0.12	0.27	
Model 3 (Depression)					0.48 (<0.001)
Covariates					
Age	0.22 (1.36)	−2.45, 2.89	0.01	0.87	
Gender	9.68 (5.19)	−0.49, 19.85	0.15	0.06	
AQ	1.04 (0.21)	0.63, 1.45	0.41	<0.001**	
Predictors					δ*R*^2^ *(p* value): 0.17 (<0.001)
* b*_0_	103.92 (3.12)	97.80, 110.04	3.66	<0.001**	
* b_1_*–YP	1.90 (0.38)	1.15, 2.66	0.48	<0.001**	
* b_2_*–C	−1.25 (0.34)	−1.92, –0.58	−0.27	<0.001**	
* b_3_*–YP^2^	0.02 (0.05)	−0.07, 0.11	0.05	0.62	
* b_4_*–YP × C	0.09 (0.04)	0.01, 0.17	0.16	0.02*	
* b_5_*–C^2^	−0.01 (0.05)	−0.16, 0.04	−0.10	0.24	

*Note.* Change scores are calculated from baseline model with covariates only. AQ: autism traits; C: caregiver; YP: young person.

* *p_adjusted_* < 0.05, **p_adjusted_ < 0.01 (using Benjamini–Hochberg corrections).

**Table 7.** Response surface for assumed similarity of autistic traits, generalised anxiety and depression scores reported by young person and caregiver in relation to total social camouflaging behaviours reported by young person in the total sample (*N* = 82): Position of first principal axis and shape of surface along the lines.

RSA	Position of first principal axis				Shape of surface along the lines								Final conclusion
					LOC				LOIC				
	*p*_10_		*p*_11_		*a*_1_		*a*_2_		*a*_3_		*a*_4_		
	Est (*SE*)	95% CI	Est (*SE*)	95% CI	Est (*SE*)	95% CI	Est (*SE*)	95% CI	Est (*SE*)	95% CI	Est (*SE*)	95% CI	
AQ	35.40 (90.57)	−142.12, 212.93	−1.06, 1.98	−4.94, 2.82	1.80*** (0.37)	1.08, 2.52	−0.05, 0.04	−0.14, 0.028	1.73* (0.79)	0.18, 3.29	−0.005 (0.051)	−0.11, 0.096	No congr eff. (*a_3_* ≠ 0)
GAD	−6.78* (3.18)	−13, –0.55	1.52** (0.50)	0.54, 2.49	1.8*** (0.53)	0.77, 2.83	−0.027 (0.12)	−0.25, 0.20	6.01*** (1.20)	3.65, 8.36	−1.14** (0.44)	−2.00, –0.27	No congr eff. (*p*_10_ ≠ 0, *a_3_* ≠ 0)
Depression	−12.90 (11.39)	−35.23, 9.43	0.44 (0.29)	−0.13, 1.01	0.65 (0.37)	−0.067, 1.37	0.053 (0.048)	−0.042, 0.147	3.16*** (0.63)	1.93, 4.39	−0.13 (0.073)	−0.27, 0.014	No congr eff. (*a_3_* ≠ 0)

LOC: line of congruence; LOIC: line of incongruence; Congr eff.: congruence effect.

* *p* < 0.05. **p < 0.01. ***p < 0.001.

### Association between assumed similarity in generalised anxiety symptom severity and social camouflaging

Figure 3(b) shows how all combinations of adolescent (X) and caregiver (Y) reports of generalised anxiety symptoms related to adolescent’s report of total social camouflaging behaviours (Z), when controlling for age, gender (male vs other gender identities) and autistic traits, and all polynomial coefficients are reported in Tables 6 and 7. Analysing the line of congruence, camouflaging was significantly higher when adolescent and caregiver reports of GAD symptoms matched at higher levels than at lower levels (*a*_1_ = 1.80, *p* < 0.001), though it was not associated with how adolescent and caregiver rated GAD symptoms matched at either midrange or extreme levels (*a*_2_ = −0.027, *p* = 0.815). Analysing line of incongruence, camouflaging was significantly greater when adolescent rated GAD symptoms exceeded caregiver ratings (*a*_3_ = 6.01, *p* < 0.001), and greater the more adolescent and caregiver ratings matched one another (*a*_4_ = −1.14, *p* = 0.01). However, when analysing the line of congruence, incongruence and first principal axis together, the RSA output did not support the congruence hypothesis. In other words, camouflaging was not greater when adolescent and parent-rated GAD symptom severity were closer to one another, as the first principal axis significantly differed from the line of congruence (*p*_10_ = −6.78, *p* = 0.03).

### Association between assumed similarity in depression symptom severity and social camouflaging

Figure 3(c) shows how all combinations of adolescent (X) and caregiver (Y) reports of depression symptoms related to adolescent’s report of total camouflaging behaviours (Z), when controlling for age, gender (male vs other gender identities) and autistic traits, and all polynomial coefficients are reported in Tables 6 and 7. Analysing the line of congruence, camouflaging was not significantly associated with how adolescent and caregiver rated depression symptoms matched at high or low levels (*a*_1_ = 0.65, *p* = 0.076), nor at extreme or midrange levels (*a*_2_ = 0.05, *p* = 0.28). Analysing line of incongruence, camouflaging was significantly higher when adolescent rated depression symptoms exceeded caregiver ratings (*a*_3_ = 3.16, *p* < 0.001). Camouflaging was not associated with the extent to which adolescent and caregiver rated depression symptom severity matched or deviated from each other (*a*_4_ = −0.13, *p* = 0.076). When analysing the line of congruence, incongruence and first principal axis together, the RSA output did not support the congruence hypothesis. In other words, camouflaging was not greater when adolescent and parent-rated depression symptom severity were closer to one another, as *a*_3_ (slope along the line of incongruence) was significantly different from 0 (*a*_3_ = 3.16, 95% CI = [1.93, 4.39], *p* < 0.001).

## Discussion

In the current study, both groups of adolescents reported greater severity in symptoms of depression and GAD compared with caregiver reports, though no rater differences were found for autistic traits (Aim 1). Across the sample, camouflaging behaviours were associated with both adolescents’ self-reports of GAD and depression symptoms, when accounting for individual differences in autistic traits (Aim 2). Using RSA, this was the first study to explore the association between camouflaging behaviours rated by adolescents and adolescent–caregiver discrepancies in reports of autistic traits, as well as depression and GAD symptoms when controlling for autistic traits (Aim 3). The strength of using RSA methodology enabled us to look at both linear, curvilinear and interaction effects between adolescent and caregiver ratings when examining each association in turn. Greater congruence between adolescent and caregiver rated autistic traits, GAD and depression symptoms was not associated with greater camouflaging scores. However, RSA parameters showed that camouflaging was greater when both adolescent and caregivers rated high levels of autistic traits and GAD symptoms, and when adolescents exceeded caregiver ratings on autistic traits, GAD and depression symptoms. By extending the literature beyond camouflaging of autistic traits, our findings are novel in showing how camouflaging can also be applied to understand adolescent and caregiver discrepancy in reporting of anxiety and depression symptoms when accounting for autistic traits.

It is important to acknowledge that the current clinical samples of autistic and non-autistic adolescents were matched on levels of social anxiety symptom severity, and this was greater than the general population and non-socially anxious autistic adolescents. Given that the social anxiety measure, SPIN, taps into core constructs such as fear of negative evaluation by others and impression management (e.g. ‘Being embarrassed or looking stupid are my worst fears’) and avoidance (e.g. ‘I avoid activities in which I am the centre of attention’), such items may show construct overlap with items from the social camouflaging scale (CAT-Q) including assimilation (‘When in social situations, I try to find way to avoid interacting with others’) and masking (‘I adjust/monitor my body language or facial expressions so that I appear interested/relaxed’).

Socially anxious adolescents who may conflate such items when interpreting and reporting behaviours due to potential construct overlap between social anxiety and camouflaging may account for why camouflaging scores in the current autism and non-autism samples are higher than previous studies in autistic and non-autistic adults and adolescents (see Supplementary Table 1, Hull et al., 2019, 2020; Bernardin, Lewis, et al., 2021; Jorgenson et al., 2020). The lack of between-group differences on self-reported social camouflaging behaviours in the current study may also reflect potential construct overlap with social anxiety symptom severity which is matched across the two groups. One recent study found that there is metric non-invariance when comparing autistic and non-autistic adults’ camouflaging behaviours using CAT-Q, suggesting possible difference in interpretation of items by group (Bureau et al., 2023). However, this study did not report between-group differences in social anxiety symptoms which may have accounted for part of the difference in camouflaging reporting. Given that there is a high degree of co-occurrence between autism and social anxiety (Spain et al., 2018), controlling for potential construct overlap between social anxiety and camouflaging by matching groups on social anxiety symptom severity is a potential strength of the current study design, though it also limits the generalisability of current findings to socially anxious adolescents irrespective of autism diagnosis.

The association between higher camouflaging scores and adolescent self-reports of autistic traits, GAD and depression symptoms may partially be accounted for by inflation due to self-report bias. Given the cross-sectional design of the study, it is not possible to infer the direction of causation as to whether increased autistic traits and mental health difficulties increased the self-perceived need to camouflage, or whether increased camouflaging maintained high levels of mental health difficulties. The pattern of results is consistent with one previous study that found a significant association between camouflaging and internalising symptoms and camouflaging in autistic and non-autistic adolescents (Bernardin, Lewis, et al., 2021), when accounting for sex and autism diagnosis. We therefore extend the previous study’s findings by replicating similar associations in a clinical sample of adolescents with significant levels of mental health difficulties and high levels of social anxiety.

For both autistic traits and GAD, we note that camouflaging was greater when adolescent and caregiver reports both reported higher levels of symptom severity rather than lower levels, suggesting that young people who experience greater autistic traits and anxiety symptoms may be both more likely to camouflage as well as being less effective in their camouflaging behaviours to hide social differences. When considering GAD, Hull et al. (2017) noted that autistic adults described using structured social techniques as a way of reducing uncertainty and an attempt to manage their anxiety in social situations. Given that intolerance of uncertainty is a key maintaining factor of anxiety over time (Dugas et al., 1998), the felt pressure to assimilate and ‘perform’ had the unintended consequence of increasing anxiety due to not knowing how effective their camouflaging behaviours may be, as well as when camouflaging was unsuccessful (Cook et al., 2021; Hull et al., 2017, 2021). A recent qualitative study with autistic adolescents noted that when internal experiences of anxiety and worry about coming across as ‘weird’ or doing something wrong drove social camouflaging behaviours, the consequences such as suppressed emotions and reduced self-confidence when unable to express one’s authentic self, further maintained anxiety over time (Chapman et al., 2022). This may partially explain why camouflaging was greater when both adolescent and caregiver rated high levels of anxiety symptoms. Autistic adolescents described how social camouflaging over time led to increased feelings of shame and poor self-image, increased loneliness and felt disconnected from others, and experienced exhaustion from cognitive and sensory overload (Chapman et al., 2022). Such findings resonate with the current study where increased social camouflaging was associated with higher GAD symptoms.

Furthermore, the finding that greater camouflaging was only associated with adolescent and caregiver reports of symptom severity at *high levels* of autistic traits and GAD is also noteworthy. Previous studies of discrepancy ratings of internalising conditions between caregivers and adolescents suggests that caregivers may conflate symptoms of autism and mental health difficulties (Kalvin et al., 2020; Storch et al., 2012), and may only be able to more readily and accurately report on clinically significant levels of anxiety that manifest through behavioural differences (Kalvin et al., 2020; Sukhodolsky et al., 2008). Therefore, for those who may have lower levels of autistic traits and/or GAD symptoms, the combined cognitive and behavioural efforts adolescents put in to hide their perceived differences may thus reduce not only autism-specific traits, but also result in the absence of more noticeable behavioural characteristics of internalising conditions (such as increased social withdrawal and avoidance), thus creating greater discrepancy between adolescents’ experience of their mental health difficulties and caregiver perceptions at lower levels of symptom severity.

By contrast, adolescents who reported higher autistic traits, GAD and depression symptoms than caregiver reports also reported greater social camouflaging behaviour. As adolescents search for their own identity and learn about one’s role in society (Erikson, 1950, 1963), they experience heightened sensitivity to peer acceptance and rejection that can heavily influence one’s emotion regulation and decision making in social situations (Blakemore & Robbins, 2012; Foulkes & Blakemore, 2018). To consider the potential influence of camouflaging on depression and anxiety symptoms later in adolescence, the unintended consequence of hiding one’s authentic self (such as one’s autistic traits) to fit in with peers may exacerbate feelings of not being understood by others. It may be that the cognitive components underpinning the decision to camouflage and turn away from one’s autism identity overlaps with the cognitive processes underlying depression and GAD. For example, camouflaging may reinforce core beliefs of one’s authentic self being unacceptable (Bernardin, Lewis, et al., 2021; Bernardin, Mason, et al., 2021; Jorgenson et al., 2020), perpetuate worries associated with ‘what if’ one is not accepted by others due to social differences, and increase feelings of shame and negative autism identity (Cooper et al., 2017, 2022) that may contribute to poorer well-being and worse mental health outcomes over time. Given that many non-autistic adolescents who experienced depression named social support from close family and friends as a main way to cope with sadness and loneliness (Farmer, 2002; McCann et al., 2012b), it is unclear to what extent social camouflaging may have interfered with caregivers’ understanding of the severity of adolescents’ depression symptoms, and in turn may affect the quantity and quality of informal support provided to adolescents.

Furthermore, autistic young people who hide their authentic self to ‘perform’ in social settings may experience an increase in emotional distance between self and others, resembling experiences of neurotypical adolescents with clinical depression (Twivy et al., 2023), who report feeling socially disconnected from others and wanting to withdraw from social interactions (McCann et al., 2012a). Altering one’s self-presentation may also parallel a sense of loss of one’s self-identity, exacerbate negative self-beliefs such as feeling inadequate (De Mol et al., 2019) and internalise blame for causing one’s own depression (Midgley et al., 2017). Non-autistic adolescents who experience depression also describe a similar fear of stigma and being judged or bullied by others similar to autistic adolescents, and hiding their symptoms of depression and sadness in social settings is often described as a way of coping (De Mol et al., 2019; McCann et al., 2012a). Therefore, experiences of depression may also exacerbate autistic adolescents’ felt sense of being different from peers, and it may be the desire to hide this difference that motivates one to engage in greater levels of camouflaging (Chapman et al., 2022).

### Limitations and future directions

A strength of the current study is in extending previous findings of the association between social camouflaging and mental health difficulties to a clinical sample of autistic and non-autistic adolescents who currently or have previously accessed CAMHS to support mental health difficulties, who were matched on elevated levels of social anxiety, with detailed reports of co-occurring mental health difficulties. When comparing raw and T-scores on the RCADS, we observed that although adolescents rated greater symptom severity via the raw scores, caregivers scored more highly above the clinical cut-off threshold when using T-scores. We chose to use raw scores in the current study for analyses as it highlighted greater individual nuances in perceived experiences between adolescents and caregivers for symptom severity ratings and given the diversity in gender identity in the current samples, translation of raw score to T-score would not hold the same validity for adolescents who identified as non-binary. Given that the current study used a largely clinical sample of autistic and non-autistic young people recruited from CAMHS, the majority were perceived by professionals to have significant levels of mental health difficulties that warranted clinical assessment and intervention, even if this may not be fully reflected by RCADS anxiety and depression scores. Our findings also mirror the relatively weaker parent–child concordance rate on the RCADS reported in autistic young people without co-occurring intellectual disability (Khalfe et al., 2023). We recommend that future studies may wish to adopt multiple measures for assessing GAD and depression when working with autistic CYP and their caregivers, allowing convergent validity to be assessed alongside rater concordance, instead of relying on a single measure for mental health difficulty characterisation.

We also highlight several limitations to consider when interpreting results. First, the sample size of the current study is of modest size, resulting in relatively low powers to detect interaction effects when looking at rater by group differences in mental health outcomes, and has limitations in completing large-sample technique such as RSA methodology. The non-autism group has high levels of autistic traits, despite not having a clinical diagnosis of autism. We acknowledge that the absence of clinical diagnosis does not exclude meeting the diagnostic cut-off for autism if formally assessed. To mitigate the impact of making conclusions based on clinical diagnosis of autism alone, we explored autistic traits as a continuum variable in the current study when examining associations with mental health difficulties in relation to social camouflaging behaviours, to ensure that individual differences in autistic traits are captured rather than diagnosis per se.

Furthermore, the current sample of non-autistic adolescents was selected for having higher levels of social anxiety matching those from the autistic group. However, this makes the sample neither representative of the general non-autistic child psychiatric population nor the general autism population, and results may not be able to fully generalise to either the general population or the broader autistic population. It should also be noted that the current clinical samples of autistic and non-autistic adolescents scored higher on both generalised anxiety and depression subscales of the self-report RCADS (means approx. 16–18 for depression; 10–12 for generalised anxiety) compared with scores in a previous validation study of using RCADS with autistic children and adolescents (aged 11–15 years) with at least one clinical diagnosis of anxiety disorder (3.49 for depression; 5.17 for generalised anxiety; Sterling et al., 2015). The complexity of clinical presentation of adolescents in the current study also extends beyond neurodevelopmental and internalising conditions such as anxiety and depression, including a myriad of other mental health difficulties. While the clinical picture of this group may limit generalisability of the current results to non-clinical populations, the complexity of psychopathology presentation may be reflective of clinical samples seen in CAMHS in the United Kingdom. Future studies may assess generalisability of the current results between social camouflaging and mental health difficulties by having a larger overall sample size, and adding a control sample of adolescents who have low levels of autistic traits and social anxiety symptoms that extend group comparisons and allow results to be more generalisable to the wider non-clinical population. Finally, the current sample is also primarily White Caucasian, and future studies may benefit from studying camouflaging in adolescents from more diverse ethnic, racial and cultural backgrounds given the potential intersectionality with other forms of stigmatised identities.

The cross-sectional nature of the current study has limited scope for inferring direction of causation between autistic traits, camouflaging, and experiences of depression and anxiety in autistic and non-autistic adolescents. Future studies may consider including a third perspective (e.g. clinician or teacher rating) of adolescents’ mental health difficulties to assess whether social camouflaging behaviours have similar or different impact on discrepancy scores across different raters and settings (e.g. home, clinical and school). The use of self-report measures of CAT-Q as the sole measure of camouflaging behaviour also has limitations as it may introduce both mono-operation and response bias. For example, the lack of between-group differences in CAT-Q measures when matching groups on social anxiety suggests there may be significant construct overlap between the CAT-Q and social anxiety, and also autistic and non-autistic adolescents may be answering items in the CAT-Q with different interpretations and situations in mind, further introducing bias when assessing the relations between CAT-Q and other covariates such as autistic traits. Future studies may also wish to draw on other ways of reporting masking (e.g. having blind raters code social interactions and rate perceived social communication skills) to address the issue of mono-operation bias and possible response bias in future studies. Understanding the impact of environmental context on adolescents’ felt need to camouflage their individual differences may directly affect the amount of more formal support offered to them in the school and clinical environment. Furthermore, the impact of online social media use that may encourage negative social comparison and influence mental health outcome may be another future direction to explore (Alhujaili et al., 2022).

### Clinical implications

In a clinical sample of autistic and non-autistic adolescents who experience mental health difficulties, the current study suggests that assessing experiences of social camouflaging may help clinicians interpret discrepancies in caregiver and adolescent reports of internalising symptoms in routine outcome measures when working with neurodiverse adolescents (Kalvin et al., 2020; Storch et al., 2012; Sukhodolsky et al., 2008). Clinicians may use the social camouflaging measure to gain insight into both cognitive and behavioural mechanisms actively employed by adolescents to hide their social differences. The current study suggests that the impact of social camouflaging may go beyond that of hiding autism-specific traits alone and be associated with mental health difficulties. Engaging in conversations with both autistic adolescents and caregivers on camouflaging may evoke insight into key cognitions and behaviours that perpetuate low self-esteem, poor self-identity and social worries that underpin depression and anxiety symptoms. By understanding motivations behind social camouflaging, clinicians and other stakeholders, including family and friends, may be better able to advocate for change in social environments to reduce autism-related stigma in society (Perry et al., 2022), and work with adolescents to develop a better understanding of their strengths and form a more positive autism identity (Cooper et al., 2017, 2022).

One qualitative study of autistic adolescents’ experience between mental health and autism noted that autistic adolescents were able to recognise when their ‘authentic’ self was present, and that masking over time became a self-fulfilling prophecy that maintained negative self-image and social anxiety (Chapman et al., 2022). It is important for clinicians to collaboratively work together with the adolescent to develop that shared understanding of how masking autistic traits may inadvertently exacerbate symptoms of mental health difficulties and vice versa, and to co-create a person-centred formulation that depicts the relationship between their mental health difficulties and autism identity. Such a formulation may form the first step to guide psychoeducation around autism and mental health difficulties at the start of psychological therapy, so that any aspects of camouflaging behaviours that may perpetuate negative core beliefs about the self and potentially negative autism identity can be actively identified. Clinicians can discuss with the adolescent both the pros and cons of maintaining such camouflaging behaviours in the short and long terms, and for clinicians to support adolescents to find the middle path between acceptance and building a positive autism identity and changing the extent to which one chooses to camouflage to reduce potential negative impact on autism identity and mental health outcomes.

## Supplemental Material

sj-docx-1-aut-10.1177_13623613241238251 – Supplemental material for Exploring the association between social camouflaging and self- versus caregiver-report discrepancies in anxiety and depressive symptoms in autistic and non-autistic socially anxious adolescents Supplemental material, sj-docx-1-aut-10.1177_13623613241238251 for Exploring the association between social camouflaging and self- versus caregiver-report discrepancies in anxiety and depressive symptoms in autistic and non-autistic socially anxious adolescents by Jiedi Lei, Eleanor Leigh, Tony Charman, Ailsa Russell and Matthew J Hollocks in Autism
