# Supplementary material for: Exploring the association between social camouflaging and self- versus caregiver-report discrepancies in anxiety and depressive symptoms in autistic and non-autistic socially anxious adolescents
Source: Autism. 2024 Mar 15;28(10):2657–74. doi: 10.1177/13623613241238251 (PMC11459877; doi:10.1177/13623613241238251)
Supplement: sj-docx-1-aut-10.1177_13623613241238251 – Supplemental material for Exploring the association between social camouflaging and self- versus caregiver-report discrepancies in anxiety and depressive symptoms in autistic and non-autistic socially anxious adolescents [file sj-docx-1-aut-10.1177_13623613241238251.docx]

**Supplementary Materials**

**Methods**

**Measures**

***Demographic information.*** Participants completed demographic questions including age, gender identity, and ethnicity. Socioeconomic status is estimated from adolescents’ report of the type of school attended, eligibility for free school meals, and caregiver report of education, employment status, and whether caregivers considered themselves and their child to be from a low socioeconomic status when compared with other people. Participants also reported co-occurring mental and physical health conditions.

***Receptive One-Word Picture Vocabulary Test, 4^th^ Edition*** *(ROWPVT-4, Martin & Brownell, 2010)* An individually administered task assessing how well each young person is able to match a spoken word (in English) to objects, actions or concepts presented in full-colour pictures using multiple choice questions. All young people began the measure on item 95 “gossiping” (i.e., for Age 14-0 +), accuracy scores were used to match participants’ basic English comprehension between the autism and non-autism groups.

***Mini-Social Phobia Inventory*** *(Mini-SPIN; Connor et al., 2001)* Initial screening for social anxiety in non-autism sample used the 3-item Mini-SPIN, where a cut-off score of 6 or greater has a sensitivity of 88.7% and specificity of 90% in detecting high levels of social anxiety symptoms.

***Autism Quotient-28*** *(AQ-28; Hoekstra et al., 2011)* Adolescents completed a self-report 28-item questionnaire (abbreviated from the full Autism Quotient and previously used in adolescent samples (Dewinter et al., 2017)) with good convergent validity (*r* = .94) to assess autistic traits. Participants rate to what extent they agree with each of autistic traits from definitely agree (1) to definitely disagree (4). In the community sample, AQ-28 has good internal consistency for the total score (α = .77-86), Social Behaviour factor (α = 0.79-0.86) and Numbers/Patterns factor (α = 0.67-0.73). A cut-off score of ≥70 has sensitivity of 0.94 and specificity of 0.91. In the present sample, internal consistency is good for the total score (α = 0.86), the Social Behaviour factor (α = 0.86), and the Numbers/Patterns factor (α = .79).

***Autism Quotient – Adolescent Version*** *(AQ-A; Baron-Cohen et al., 2006)* Caregivers completed the 50-item adolescent version of autism quotient to report autistic traits in their young person. Caregivers rated to what extent each statement applied to their young person from definitely agree to definitely disagree, giving a sum total score out of 50. In the community sample, AQ-A has good internal consistency for the total (α = .79) and all subscales scores (α = 0.66 – 0.88), with a suggested cut-off score of >30. In the present sample, internal consistency is good for the total (α = 0.85) and all subscale scores (α = 0.69 - 0.77) except for details subscale (α = 0.42).

***Social Phobia Inventory*** *(SPIN; Johnson et al., 2006)* Adolescents completed a self-report 17-item questionnaire assessing symptoms of social anxiety. Participants rate how much they were bothered by each of the symptoms in the past week from not at all (0) to extremely (4). SPIN has good internal consistency for the total score (α = 0.92). In community adolescent samples, a cut-off score of 21 has good sensitivity (68.3%) and specificity (81.4%). In the current sample, SPIN showed good internal consistency (α = 0.92).

***Camouflaging Autistic Traits Questionnaire*** *(CAT-Q; Hull et al., 2019)* Adolescents completed a self-report 25-item questionnaire assessing social camouflaging behaviours, including subscales assessing masking, compensation and assimilation. Participants rate the extent to which they agree with each statement of a camouflaging behaviours on a scale of 1 (strongly disagree) to 7 (strongly agree). In autistic and neurotypical adult community samples, CAT-Q has good internal consistency for the total score (α = 0.94), as well as for the subscales (α = 0.85-0.92). In the current sample, CAT-Q total showed good internal consistency (α = 0.93), as well as all subscales (α = 0.80 – 0.91).

***Revised Children’s Anxiety and Depression Scale – Depression, Generalised Anxiety, and Social Phobia subscales*** *(RCADS-DEP, RCADS-GAD; RCADS-SocP; Baron et al., 2021)* Both adolescents and caregivers completed this short form to rate how often each statement applies to the young person from 0 (never) to 3 (always), with 10 items focused on symptoms associated with low mood (α = 0.78) and 6 items associated with generalised anxiety (α = 0.83). In the current sample, adolescents and caregivers showed good internal consistency for depression (α = 0.88 for both) and generalised anxiety (α = 0.87, 0.86 respectively) subscales. T-scores >65 is seen as borderline clinical threshold, and T-score ≥70 seen as above clinical threshold (Esbjørn et al., 2012). Good internal consistency for social phobia subscale rated by caregivers was also found (α = 0.90).

**Analyses**

All statistical analyses were completed in SPSS v.28, and Response Surface Analysis was completed in *R* using RSA package (Schönbrodt & Humberg, 2023). All participants completed all measures with no missing data. First, descriptive statistics were computed for both caregiver and adolescent reported demographic variables. Between-group differences in age for caregiver and adolescent were explored using independent sample t-tests. Between-group differences in gender, ethnicity, caregiver employment and education status, and adolescent’s eligibility for free school meals were explored using chi-squared tests.

Second, four independent two-way ANOVAs were completed to assess differences in ratings of autistic traits, symptoms of depression, generalised anxiety, and social anxiety by rater (adolescent vs. caregiver), and by diagnostic group (autism vs. non-autism). Post-hoc analyses were completed to assess any significant main and interaction effects. We used Benjamini-Hochberg procedure to control for false discovery rate and multiple comparisons, and report adjusted *p* values. Given that self and care-giver measures of social anxiety and autistic traits used different scales, standardised z-scores were calculated for each of the scales based on the sample mean for adolescents and caregivers for subsequent analyses to allow data to be compared across raters and groups. To assess inter-rater agreement between adolescents and caregivers, we generated Bland-Altman plots to visually depict systematic trends in informant agreement across the range of scores on the RCADS for depression and generalised anxiety subscales, and tested whether the average mean score of adolescent and caregiver for each subscale was associated with the difference score (Bland & Altman, 1995).

Third, we completed partial correlations (controlling for age, gender – using male versus other gender identities as binary dummy coding) to explore the association between social camouflaging behaviours and individual differences in symptom severity for autistic traits, generalised anxiety and depression as reported by adolescents. We used Benjamin-Hochberg procedure to control for false discovery rate and multiple comparisons, and report adjusted *p* values.

Fourth, we completed response surface analysis (RSA) to explore to what extent assumed similarity in ratings of autistic traits, generalised anxiety, and depression severity ratings by adolescents and their caregivers are associated with total social camouflaging scores. For each model, we first centred each predictor on the midpoint of the questionnaire. To aid clinical interpretation, we outline how the midpoint of each scale relates to clinical cut-off thresholds for:

1. Autism Quotient-50 (caregiver): midpoint 25 falls below cut-off score of >30
2. Autism Quotient-28 (adolescent): midpoint 56 falls below cut-off score of ≥70
3. RCADS-GAD: midpoint 9 falls below clinical cut-off T-score of ≥70 for all adolescents, and for caregivers of young people aged 14-15 years old. Midpoint exceeds clinical cut-off T-score for caregiver ratings of young people aged 16-18 years.
4. RCADS-DEP: midpoint 15 falls below clinical cut-off T-score of ≥70 for adolescent boys aged 14-15 years, and adolescent girls aged 16-18 years. Midpoint exceeds clinical cut-off T-score for caregiver ratings for all age groups, and for adolescent girls aged 14-15 years, and adolescent boys aged 16-18 years.

We included the following predictors: 1) adolescent’s self-report symptom severity (X), 2) caregiver’s report of adolescent’s symptom severity (Y), 3) the quadratic term of adolescent’s self-report (X^2^), 4) the interaction between adolescent and caregiver report (X*Y), 5) the quadratic term of caregiver’s report (Y^2^). We added age and gender (male versus other gender identities) as covariates for RSA on autistic traits, and added age, gender, and autistic traits for RSA on generalised anxiety and depression symptom severity. We chose the RSA approach over hierarchical linear regressions that predict social camouflaging behaviour by directly using discrepancy scores between adolescent and caregiver reports, because the former offers more nuanced information on the linear and curvilinear relationship at *different levels* of both *matches and mismatches* between different predictors with the outcome variable (Barranti et al., 2017). The line of congruence provides information about how agreements between adolescent and caregiver ratings at different levels of symptom severity is associated with social camouflaging behaviours. The line of incongruence provides information about how disagreements between adolescent and caregiver ratings at different levels of symptom severity is associated with social camouflaging behaviours. Finally, combined with the first principal axis, all information combined can inform whether the congruence hypothesis is satisfied by using the steps laid out by Humberg et al. (2019).

Supplementary Materials - Table 1. Comparison of scores from current study to previous studies in autistic and non-autistic samples:

1. CAT-Q Scores (Social Camouflaging) in adolescent and adult samples

|  |  | | Autism | | | Non-Autism | | |
| --- | --- | --- | --- | --- | --- | --- | --- | --- |
|  | Current study | Hull et al. (2019) | | Hull et al. (2020) | Jorgenson et al. (2020) / Bernardin et al. (2021)^1^ | Current study | Hull et al. (2019) | Jorgenson et al. (2020) / Bernardin et al. (2021)^1^ |
| Sample size (n female) | 43 (25) | 200 | | 58 (29) | 78 (23) | 39 (27) | 202 | 62 (35) |
| Mean age (years) | 15.70 | ~37.02^2^ | | 14.48 | 15.03 | 15.97 | ~37.02^2^ | 15.31 |
| Compensation (M) | 35.86 | 39.78 | | 35.29 | 33.60 | 31.82 | 26.01 | 32.92 |
| Masking (M) | 36.26 | 36.4 | | 35.93 | 31.29 | 39.69 | 34.32 | 38.26 |
| Assimilation (M) | 39.91 | 42.32 | | 33.82 | 33.56 | 38.00 | 34.4 | 26.21 |
| CAT-Q Total (M) | 112.02 | 119.75 | | 105.03 | 99.46 | 109.51 | 87 | 98.39 |

*Note.* ^1^Same participant sample from SPARK study. ^2^Mean age for combined autism and non-autism sample in study. CAT-Q = Camouflaging Autistic Traits Questionnaire.

1. Social anxiety scores in adolescent samples

|  |  | | Autism | | | Non-Autism | | |
| --- | --- | --- | --- | --- | --- | --- | --- | --- |
|  | Current study – SPIN total | Wood et al. (2022) – SPIN total | | Cooper et al. (2022) – SAS-A | Lei et al. (2021) – SAS-A | Current study – SPIN total | Ranta et al. (2007) – SPIN total | Lei et al. (2021) – SAS-A |
| Sample size (n female) | 43 (25) | 72 (32) | | 121 (36) | 145 (43) | 39 (27) | 5252 (2658) | 267 (213) |
| Mean age (years) | 15.70 | 17.91 | | 17.60 | 17.59 | 15.97 | 15.30 | 18.28 |
| Social Anxiety (M; SD) | 37.49  (15.40) | 23.88  (13.44) | | 61.66  (13.93) | 59.99  (13.97) | 37.59  (13.95) | 12.2  (8.70) | 56.02  (11.94) |

*Note*. SPIN = Social Phobia Inventory (range: 0-68; clinical cut-off score is 24); SAS-A = Social Anxiety Scale for Adolescents (range: 18-90; clinical cut-off score is 50). All comparative samples were recruited from general population and not from clinical services.
